# Topics of the Month

**Published:** 1893-08

**Authors:** 


### TOPICS OF THE MONTH.

ERRORS in school books are of frequent occurrence, and pertain to almost every author and all editions. These errors involve almost every question relating to the history of our country. In school books are published absurd theories about politics, currency, capital, labor, free trade, traffic, and all of the important questions of political economy, that are misleading to the young mind and even puzzling to the more mature individual. It is, therefore, important to correct these evils at once, and we are pleased to note that Col. Albert A. Pope, of Boston, is endeavoring to do this. He has already, that is to say, over a year ago, instituted one search, and he is now starting out upon another. As a stimulant to the search, he offers to award one of the best pneumatic tire Columbian safeties, 1893 pattern, price, $150, to each of five persons who shall send him the greatest number of errors which shall be determined to be errors by the publishers and authors of books in which they occur, or by a board of examiners which he may appoint. These errors must be received prior to September 1, 1893.

For conditions upon which the awards will be given, and other information, communications should be addressed to the Educational Department, Pope Manufacturing Co., Boston, Mass.

---

THE Jennie Casseday Infirmary for Women will be closed for improving and enlarging the building during the month of August. It will be re-opened for reception of patients September 1st. Dr. L. S. McMurtry is the surgeon in charge, and he has been doing a remarkable series of successful abdominal sections during the infirmary-year just closed.

